# Mangrove tree (*Avicennia marina*): insight into chloroplast genome evolutionary divergence and its comparison with related species from family Acanthaceae

**DOI:** 10.1038/s41598-021-83060-z

**Published:** 2021-02-11

**Authors:** Sajjad Asaf, Abdul Latif Khan, Muhammad Numan, Ahmed Al-Harrasi

**Affiliations:** 1 Natural and Medical Sciences Research Center, University of Nizwa, Nizwa, 616 Oman; 2 Department of Biology, University of North Carolina at Greensboro, 363 Sullivan Science Building, NC, 27402-6170 USA

**Keywords:** Bioinformatics, Restoration ecology, Wetlands ecology, Genome, Sequencing

## Abstract

*Avicennia marina* (family Acanthaceae) is a halotolerant woody shrub that grows wildly and cultivated in the coastal regions. Despite its importance, the species suffers from lack of genomic datasets to improve its taxonomy and phylogenetic placement across the related species. Here, we have aimed to sequence the plastid genome of *A. marina* and its comparison with related species in family Acanthaceae. Detailed next-generation sequencing and analysis showed a complete chloroplast genome of 150,279 bp, comprising 38.6% GC. Genome architecture is quadripartite revealing large single copy (82,522 bp), small single copy (17,523 bp), and pair of inverted repeats (25,117 bp). Furthermore, the genome contains 132 different genes, including 87 protein-coding genes, 8 rRNA, 37 tRNA genes, and 126 simple sequence repeats (122 mononucleotide, 2 dinucleotides, and 2 trinucleotides). Interestingly, about 25 forward, 15 reversed and 14 palindromic repeats were also found in the *A. marina*. High degree synteny was observed in the pairwise alignment with related genomes. The chloroplast genome comparative assessment showed a high degree of sequence similarity in coding regions and varying divergence in the intergenic spacers among ten Acanthaceae species. The pairwise distance showed that A. *marina* exhibited the highest divergence (0.084) with *Justicia flava* and showed lowest divergence with *Aphelandra knappiae* (0.059). Current genomic datasets are a valuable resource for investigating the population and evolutionary genetics of family Acanthaceae members’ specifically *A. marina* and related species.

## Introduction

Mangroves are woody shrub and tropical plants that grow well in the inter-tidal zones of tropical to sub-tropical latitudes^1^. Globally, mangrove covers about 200,000 km^2^ area^2,3^ and belongs to family Acanthaceae that comprises of more than 400 species of polyphyletic group of trees^4^. These trees possess unique physio-morphological adaptations and tolerance against hypersaline environment, tidal cycles, and soil chemistry^5–7^. Importantly, mangroves serve as a hub of exponential ecological resource for habituating diverse marine life by providing a protective sanctuary to bread, shelter and grow living organisms and as a sink for continued carbon emissions. However, overexploitation of mangroves for wood and environmental pollution have drastically affected the tree population as well resulted in loss of genetic diversity^8^. The evolutionary history, tolerance-based mechanisms, genetic divergence and sub-speciation are the key aspects that require in-depth studies.

Among mangroves, *Avicennia marina* (Forssk., Vierh., gray mangroves) is one the keystone species of the genus and well-distributed species across different latitudes mostly through the dispersal of diasporas by sea and wind^9,10^. Gray mangroves have been divided into categories based on their habitat and importance to the community structure^11^. Currently, at least three sub specific or allopatric varieties of *A. marina* viz. var. *australasica*,* euclayptifolia* and marina have been categorized to date^12^. The true mangroves are further distinguished as major and minor mangroves where variations also exist in habitat exclusively in aquatic and terrestrial or both^13^. However, plant scientists did not reach a consensus classification^14,15^. DNA based molecular markers system are available to discriminate among population and species and perform phylogenetic analysis^16–18^, however, current advancement of next-generation sequencing methods can help to understand genome architecture, structure, content, divergence and evolutionary history that could solve many key questions related to population structure and taxonomy^19–23^.

In this case, the chloroplast genome offers a highly conserved sequence due to uni-parent inheritance, haploid, and non-recombinant nature^24,25^. To date, about 4000 chloroplast genomes have been sequenced^26^. The chloroplast genome size for angiosperm is around 110-165 kb containing unigenes from 90 to 110^27,28^. Almost all angiosperm chloroplast genomes consist of 4 regions viz. large single-copy region (LSC), followed by inverted repeats (two complementary to each other) and a single small copy region (SSC)^29^. Due to these reasons, chloroplast genomes sequences are most recommended and used for evolutionary studies, barcoding and phylogenetic analysis^30,31^. In case of *A. marina*, though recently Frilis et al.^32^ reported the first whole-genome data with genome size of 456 million base pair, however, still the sequence datasets for Acanthaceae (± 400 species) is only 15 organelle genomes. The genus suffers from lack of a comprehensive comparative genomic assessment to understand the phylogenomic and evolutionary history of *A. marina* and related species. Hence, in the current study we aimed to sequence *A. marina* growing in Oman and perform comparative chloroplast genome analysis with *Andrographis paniculate*,* Aphelandra knappiae*, *Clinacanthus nutans*, *Echinacanthus attenuates*,* Echinacanthus lofouensis*, *Echinacanthus longipes*,* Echinacanthus longzhouensis*,* Justicia flava*,* Justicia leptostachya* and *Strobilanthes cusia*species from Acanthaceae family.

## Results

### Chloroplast genome structure of *A. marina*

**Figure 1:**
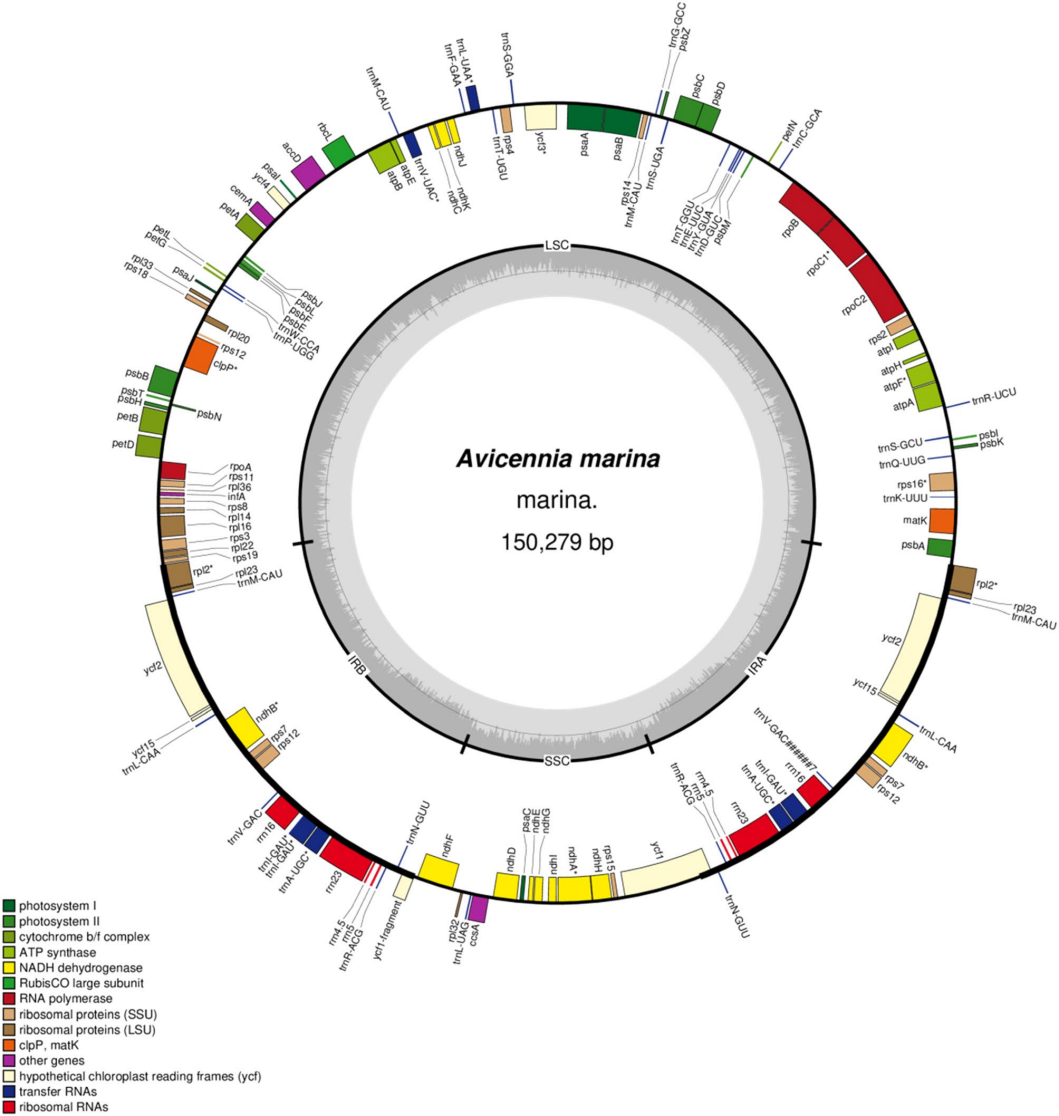
Gene map of the *Avicennia marina* chloroplast genome. Genes drawn inside the circle are transcribed clockwise, and those outside the circle are transcribed counterclockwise. The stars indicate the intron-containing genes. Genes belonging to different functional groups are colour-coded. The darker grey in the inner circle corresponds to GC content, and the lighter grey corresponds to AT content.

The length of complete chloroplast (cp) genome of *A. marina* is 150,279 bp and it exhibits a typical quadripartite structure with a pair of inverted repeats 25,117 bp that separate a large single-copy region 82,522 bp and a small single copy region 17,523 bp (Fig. 1; Table 1). The cp genome contains overall GC contents of 38.6%. We identified 22 introns containing genes (14 protein coding genes and 7 tRNA genes), among these genes 19 genes are single intron and two genes (*ycf3* and *clpP*) contain two introns. The largest intron was found in the gene *trnK-UUU* with 2,414 bp in size and the smallest intron with 486 bp in gene *trnL-UAA*. Additionally, the largest exon was found in the gene *ndhB* with 775 bp and the smallest exon was found in *petB* genes and was only 6 bp in size. Among the coding genes, *rps12* was unequally divided, with its 5′ exon being located in the LSC region and one copy of the 3′ exon and intron being located in each of the IR regions, as in other angiosperms^33,34^.

**Table 1:** Genomic details and statistics of 11 mangrove species of the family Acanthaceae.

	*A. marina*	*A. paniculata*	*A. knappiae*	*C. nutans*	*E. attenuatus*	*E. lofouensis*	*E. longipes*	*E. longzhouensis*	*J. flava*	*J. leptostachya*	*S. cusia*
Size (bp)	150,279	150,249	152,457	151,669	152,672	151,333	152,644	152,385	150,888	149,227	144,133
Overall GC contents	38.6	38.3	38.5	38.4	38.3	38.7	38.6	38.6	38.2	38.2	38.2
LSC size in bp	82,522	82,402	83,861	83,502	83,568	82,527	83,833	79,203	82,970	82,114	92,666
SSC size in bp	17,523	17,110	17,888	17,435	17,739	17,397	17,388	17,571	16,893	16,975	17,811
IR size in bp	25,117	25,397	25,354	25,366	25,662	25,688	25,691	25,434	25,500	25,069	17,328
Protein coding regions size in bp	79,321	78,549	78,678	68,634	78,930	62,904	72,939	79,203	79,833	78,024	71,370
tRNA size in bp	2839	2715	2793	2709	2645	2716	2646	2715	2967	2793	2800
rRNA size in bp	9054	9054	9052	9052	9058	9186	9186	9058	9048	9052	9078
Number of genes	132	132	131	131	129	131	131	132	137	131	129
Number of protein coding genes	87	87	86	83	86	85	87	87	88	86	84
Number of rRNA	8	8	8	8	8	8	8	8	8	8	8
Number of tRNAs	37	37	37	36	35	36	35	36	39	37	37
Genes with introns	15 + 7	14 + 8	14 + 8	15 + 8	15 + 7	15 + 8	15 + 7	15 + 8	13 + 9	14 + 8	14 + 8

The protein coding genes included larger and smaller subunit proteins such as *rpl2*,* 14*,* 16*,* 20*,* 22*,* 23*,* 32*,* 33*,* 36* and *rps2*,* 3*,* 4*,* 7*,* 8*,* 11*,* 12*,* 14*,*15 *,*16*,* 18*,* 19* respectively (Table 2). Numerous groups of photosynthetic genes were also annotated including *photosynthesis I*, *photosynthesis II*, *Cytochrome b6/f complex*, ATP synthase and *rubisco* as shown in Table 2. The genome also contains a total of 132 genes including 87 protein coding genes, 8 ribosomal rRNA genes and 37 transfer tRNA genes. The total size for the protein coding region was found 79,321 bp (52% of the cp genome), for the ribosomal RNA (rRNA) the size is 9,054 bp (6.02%) and for the transfer RNA (tRNA) it is 2,839 bp (1.88). GC contents in the coding region of the chloroplast genome was found as 43.4%. Similarly, 36.8, 32.7, 43.6, 53.1, 55.3 and 38.4% of the GC contents were found in LSC, SSC, IR, tRNA, rRNA and protein coding genes, respectively. The GC content in the protein coding genes at first, the second and third position of codon were 46.06, 37.87 and 32.25% respectively as shown in Table 3. The AT distribution in chloroplast genome of *A. marina* was found 36.7%. The AT in LSC, SSC, IR, tRNA, rRNA and protein coding genes were 63.2, 67.3, 56.4, 46.9, 44.8 and 61.6% respectively. Within the protein coding genes, the AT/U was found 54.8, 62.1 and 67.8% on first, second and third position of codons as shown in Table 3. In *A. marina* the most common amino acid was found as leucine (10.7%) and the least common amino acid was cysteine (1.2%).

**Table 2:** Genes annotated in the chloroplast genome of *A. marina*.

Category	Group of genes	Name of genes
Self-replication	Large subunit of ribosomal proteins	*rpl2*, *14*, *16*, *20*, *22*, *23*, *32*, *33*, *36*
	Small subunit of ribosomal proteins	*rps2*,* 3*,* 4*,* 7*,* 8*,* 11*,* 12*,* 14*,*15 *,* 16*,* 18*,* 19*
	DNA dependent RNA polymerase	*rpoA*,* B*,* C1*,* C2*
	rRNA genes	*rrn 4.5*,* rrn 5*,* rrn 16*,* rrn23*
	tRNA genes	*tRNA-UGC*,* trnC-GCA*,* trnD-GUC*,* trnE-UUC*,* trnF-GAA*,* trnfM-CAU*,* trnG-UCC*,* trnI-GAU*,* trnK-UUU*,* trnL-CAA*,* trnL-UAA*,* trnL-UAG*,* trnM-CAU*,* trnN-GUU*,* trnP-UGG*,* trnQ-UUG*,* trnR-ACG*,* trnR-UCU*,* trnS-GCU*,* trnS-GGA*,* trnS-UGA*,* trnT-GGU*,* trnT-UGU*,* trnV-GAC*,* trnV-UAC*,* trnW-CCA*,* trnY-GUA*
Photosynthesis	Photosystem I	*psaA*,* B*,* C*,* I*,* J*,
	Photosystem II	*psbA*,* B*,* C*,* D*,* E*,* F*,* H*,* I*,* J*,* K*,* L*,* M*,* N*,* T*,* Z*
	Cytochrome b6/f complex	*petA*,* B*,* D*,* G*,* L*,* N*
	ATP synthase	*atpA*,* B*,* E*,* F*,* H*,* I*
	Rubisco	*rbcL*
Other genes	Maturase	*matK*
	Protease	*clpP*
	Envelop membrane protein	*cemA*
	Subunit Acetyl- CoA-Carboxylate	*accD*
	c-type cytochrome synthesis gene	*ccsA*
Unknown	Conserved Open reading frames	*ycf1*,*2*,* 3*,* 4*,* 15*

**Table 3:** Base composition of the *A. marina* and other related species cp genome.

	Genome	LSC	SSC	IR	tRNA	rRNA	Protein Coding genes	1st position	2nd position	3rd position
**T/U**										
*A. marina*	24.9	32.5	33.7	28.2	25.2	18.8	31.1	23.8	33.0	36.5
*A.paniculata*	31.2	32.4	34	28.4	25.4	18.8	31.4	23.8	32.6	37.7
*A.knappiae*	31.1	32.3	33.5	28.1	25	18.7	31.3	23.7	32.5	37.7
*C.nutans*	31.1	32.3	34	28.1	25.2	18.7	31.6	23.9	32.6	38.1
*E. attenuates*	31.1	32.3	33.9	28.3	24.9	18.7	31.4	23.8	32.6	37.7
*E. lofouensis*	31	32.1	33.8	28.2	25	19	31.4	23.4	32.9	37.8
*E. Longipes*	31	32.1	33.8	28.2	24.9	19	31.3	23.7	32.5	37.7
*E.longzhouensis*	31	32.1	33.8	28.1	24.9	18.7	31.2	23.8	32.5	37.4
*J. flava*	31.2	32.4	33.8	28.3	25.1	18.7	31.5	23.9	32.7	38.0
*J. leptostachya*	31.2	32.4	33.5	28.4	25	18.7	31.5	23.9	32.6	37.9
*S.cusia*	31.3	32.3	33.9	27.3	25	18.7	31.4	23.6	32.9	37.8
**C**										
*A. marina*	22.9	18.9	17.1	22.5	23.7	23.7	18	19.0	20.0	14.9
*A.paniculata*	19.5	18.7	16.7	22.5	24.2	23.7	17.9	19.1	20.4	14.1
*A.knappiae*	19.6	18.7	17.1	21	24.1	23.8	18	19.1	20.5	14.2
*C.nutans*	19.5	18.7	16.8	20.9	24.1	23.7	18.1	19.1	20.9	14.1
*E. attenuatus*	19.5	18.6	17	20.9	24	23.7	17.8	19.0	20.4	14.1
*E. lofouensis*	19.7	19	17.2	21	24.1	23.4	17.8	18.5	20.7	14.2
*E. Longipes*	19.6	18.8	17.2	21	24.1	23.4	18.2	19.3	20.8	14.5
*E.longzhouensis*	19.7	18.9	17.1	21	24.2	23.6	18	19.1	20.5	14.5
*J. flava*	19.5	18.6	16.7	20.9	23.6	23.7	17.8	19.0	20.5	13.9
*J. leptostachya*	19.4	18.6	16.9	22.4	24.5	23.7	17.8	19.1	20.5	13.8
*S.cusia*	19.5	18.7	17	24.2	23.6	23.6	17.9	18.8	20.6	14.2
**G**										
*A. marina*	20.5	17.9	15.6	21.1	29.4	31.6	20.4	26.1	17.9	17.3
*A.paniculata*	18.8	17.7	15.1	21	28.8	31.6	20.3	26.5	17.9	16.5
*A.knappiae*	18.9	17.8	15.7	22.6	29.1	31.7	20.5	26.8	18.0	16.6
*C.nutans*	18.8	17.7	15.6	22.6	28.9	31.6	20.9	27.4	18.3	16.9
*E. attenuatus*	18.8	17.7	15.5	22.5	29.1	31.8	20.5	26.8	17.9	16.8
*E. lofouensis*	19	18	15.7	22.5	29	31.5	20.7	27.8	18.4	16.0
*E. Longipes*	19	18	15.7	22.5	29.1	31.5	20.9	27.3	18.3	17.2
*E.longzhouensis*	19	17.9	15.7	22.6	29	31.8	20.6	26.8	18.0	16.9
*J. flava*	18.8	17.6	15.5	22.5	29.3	31.6	20.4	26.6	17.9	16.6
*J. leptostachya*	18.7	17.6	15.6	21	28.8	31.6	20.4	26.7	17.9	16.7
*S.cusia*	18.7	17.9	15.3	21.5	29.5	31.8	20.5	27.3	17.9	16.3
**A**										
*A. marina*	31.8	30.7	33.6	28.2	21.7	26	30.5	31.0	29.1	31.3
*A.paniculata*	30.5	31.2	34.2	28.2	21.7	26	30.4	30.6	29.1	31.6
*A.knappiae*	30.5	31.1	33.8	28.2	21.8	25.9	30.3	30.3	29.0	31.5
*C.nutans*	30.5	31.2	33.6	28.4	21.7	26	29.5	29.6	28.1	30.8
*E. attenuatus*	30.6	31.4	33.6	28.3	22	25.8	30.3	28.5	29.2	31.4
*E. lofouensis*	30.3	31	33.2	28.3	21.9	26	30.1	30.3	28.0	32.0
*E. Longipes*	30.4	31.1	33.3	28.3	21.8	26	29.5	29.7	28.3	30.6
*E.longzhouensis*	30.4	31.1	33.4	28.2	21.8	25.8	30.2	30.4	29.0	31.2
*J. flava*	30.6	31.4	34	28.4	22	26	30.3	30.5	28.9	31.6
*J. leptostachya*	30.7	31.4	34	28.2	21.8	26	30.3	30.4	29.0	31.6
*S.cusia*	30.5	31.2	33.8	27	21.9	25.9	30.2	30.2	28.5	31.7

### Comparative analysis of *A. marina* cp genome with related species

In order to further analyze the characteristics of the *A. marina* chloroplast genome, its assembled genome was compared with the chloroplast genomes of 10 other species of the same family. The results revealed that *A*. *marina* (150,279 bp) cp genome size is slightly bigger as compared to *A. paniculata* (150,249 bp), *J. leptostachya* (149,227 bp) and *S. cusia* (144,133 bp). However, it has a slightly smaller cp genome size when compared to the species *A. knappiae*,* C. nutans*,* E. attenuates*,* E. lofouensis*,* E. longipes*,* E. longzhouensis* and *J. flava* (Table 1)*.*

The GC contents of *A. marina* (38.6%) were found almost similar for species (*A. knappiae*,* E. longipes*,* E. lofouensis* and *E. longzhouensis*), however GC contents were found higher when compared to the species *A. paniculata* (38.3%), *C. nutans* (38.4%), *E. attenuates* (38.3%), *J. flava* (38.2%),* J. leptostachya* (38.2%) and *S. cusia* (38.2%)*.* The number of genes, rRNA, tRNA, SSC size and the number of protein coding regions were found almost similar in these studied cp genomes Table 1. The LSC analysis showed that it is almost similar in all the species except *E. longzhouensis* where the size was found slightly smaller (79,203 bp) but significantly higher in *S. cusia* (92,666 bp) when compared to *A. marina* (82,522 bp).

The synteny of *A. marina* cp genome with ten other species from Acanthaceae was analyzed by mVISTA. Gene divergence was determined by determining pairwise alignment of *A. marina* with related species. *A. marina* chloroplast genome was used for reference to determine variation and sequence identity in the chloroplast genomes of related species. The results showed high sequence similarities among the cp genomes of several species, especially in protein-coding and IR regions (Figure S1).

**Figure 2:**
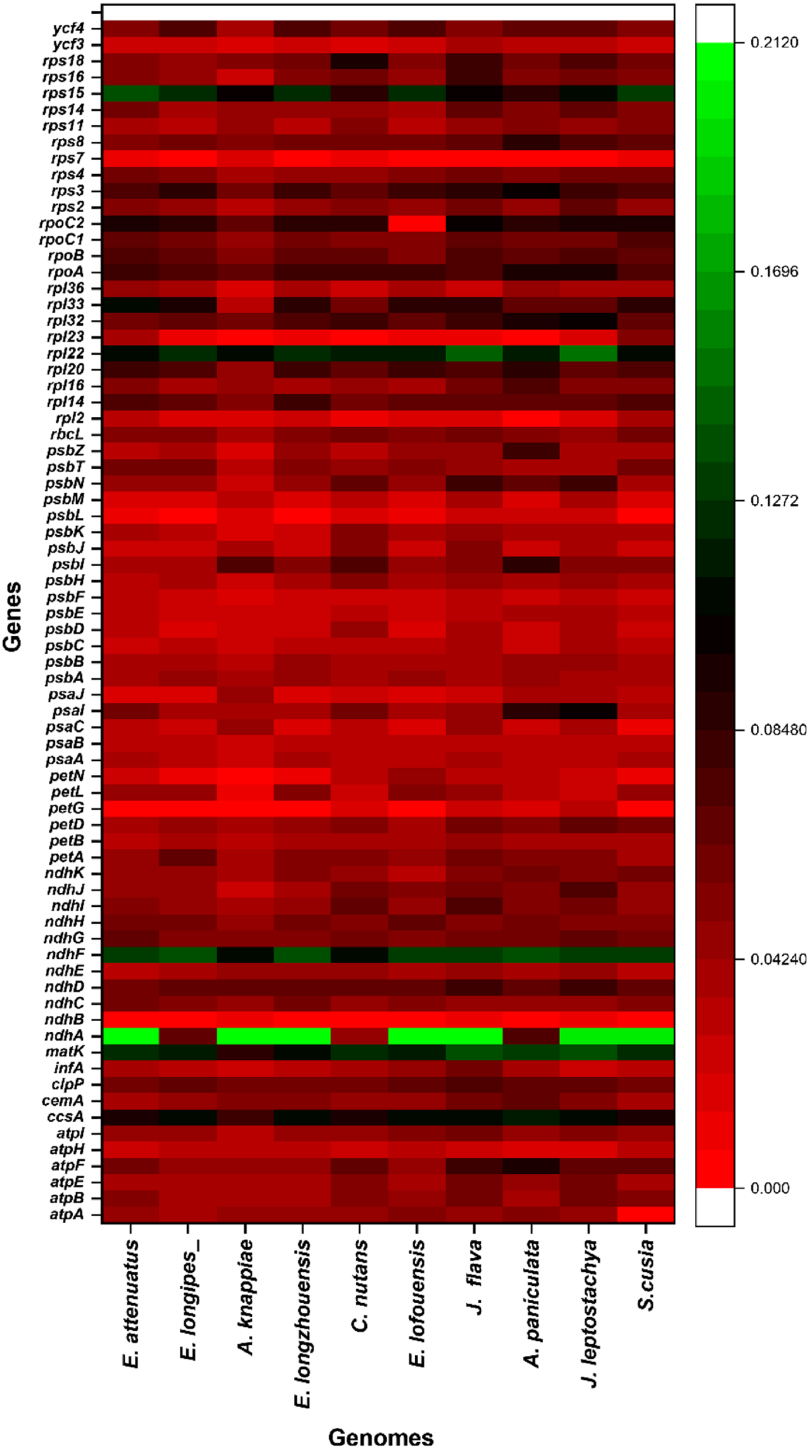
Heatmap of pairwise gene divergence in the chloroplast genome sequence of *A. marina.* The highly divergent genes are represented with light and green color depending on the divergence frequency.

For example, the intergenic regions between *psbA-matK*,* rps16-psbL*,* atpA-atpF*,* atpH-atpI*,* rpoC1-rpoB*,* psbE-petG*,* petN-psbM*,* psbD-rps14*,* ycf3—rps4*,* atpE-rbcL*,* accD-ycf4*,* psbL-petL*,* clpP-psbN*, *petD-rpl36*,* rpoA-rps11*,* rpl22-rps8*,* rpl16-rps3*,* ycf15-ndhB*,* rps19-rpl23*,* ndhF-ccsA*,* ndhD-psaC*,* ndhI-ndhG*,* ndhA-ycf1* and *ndhB-ycf2* were found highly divergent. Besides these intergenic regions some divergence was observed in protein-coding genes (*rps16*,* rpoC1*,* rpoC2*,* atpA*,* matK*,* atpF*,* clpP*,* rps12*,* psbN*,* psbB*,* psbT*,* psbH*,* petB*,* accD)* in LSC region, *(petD*,* rpl16*,* rpl22*,* rpl36*,* rps3*,* rps8ycf2)* in IR region and *(ycf1*,* ycf15*,*ndhA*,* ndhB*,* ndhF and ndhH*) in SSC region*.* The gene divergence was found more prominent in LSC and SSC region as compared to the IR region. In pairwise sequence divergence analysis, *A. marina* exhibited highest divergence (0.084) with *J. flava* and showed lowest divergence with *A. knappiae* (0.059) (Supplementary Data 1). The most divergent genes were *rpl22 (J. leptostachya* (0.149), rps15 in *E. attenuates* (0.135), *ndhF A. paniculate* (0.139), *E. lofouensis* (0.212), and *matK J. leptostachya* (0.139) (Fig. 2). Similarly, lowest pairwise divergence was found in genes such as, *ndhB* (0.003) in *E. longzhouensis*, *psbL* (0.009) in *E. lofouensis*, *petN* (0.011) in *E. longipes. psaJ* (0.015) in *E. attenuates*.

### Simple sequence repeat (SSR) analysis of A. marina cp genome

**Figure 3:**
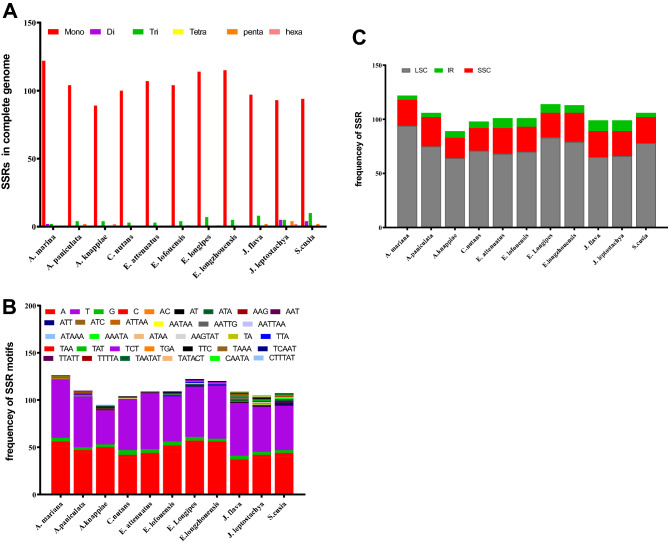
Simple sequence repeats (SSRs) analysis in the chloroplast genome of *A. marina*. (**A**) Numbers of SSR types in complete chloroplast genome, (**B**) Number of SSRs in LSC, IR and SSC regions. (**C**) frequency of SSR motifs.

We determined SSRs in the cp genome of *A. marina* as well as in the cp genomes of the other ten related species (Fig. 3A). A total of 126 SSRs were found in the chloroplast genome of *A. marina.* Among the predominant SSRs nucleotides such as mononucleotide, dinucleotide, trinucleotide, etc., the mononucleotide SSR was found the most abundant not only in *A. marina* but also in the other species of the current study. In this report the presence, distribution and types of SSRs were studied (Fig. 3B). The SSRs in the IR, LSC and SSC regions of the chloroplast genomes of above-mentioned species have been analyzed and were found distributed such as mono, di and trinucleotidies. However, most of the SSRs in these species were found mononucleotides in the IR region. For example, mononucleotides SSRs in *A. marina*,* A. paniculata* and *S. Cusia* were 4, in the *A. knappiae* and *C. nutans*, the number of SSRs were found 6, in *E. lofouensis*, *E. longipes*, *J. flava* and *J. leptostachya* have 8 SSRs as mononucleotides, in the *E. attenuates* and *E. longzhouensis* 9 and 7 mononucleotide SSRs were found. Similarly, *J. flava* contain 2 dinucleotide SSRs in the inverted repeat regions, in the IR region the *J. leptostachya* contain 1 penta and hexa nucleotide SSR each. SSRs in LSC region were found mostly as mononucleotide and trinucleotide in all the above-mentioned species. For example, In the *A. marina* 90 mononucleotides SSRs were found. In all other 10 species, in LSC, mononucleotide SSRs were found in the range of 56 to 77. For example, the *E. longipes* contain 2nd higher number (77 mononucleotide SSRs) after *A. marina* in LSC region. Similarly, the *E. longzhouensis* has 76 mononucleotide SSRs, *A. paniculata* 70, *C. nutans* 68, E. *attenuates* and *S. Cusia* 65, A. *knappiae* and *J. flava* 59 and *J. leptostachya* 56 SSRs in LSC region. In some species dinucleotide SSRs were found in LSC region such as 2 in *A. marina*, 1 in *J. flava*, 5 in *J. leptostachya* and 4 in *S. cusia*. Similarly, in LSC region *A. marina* contain 2 trinucleotides SSRs while the highest trinucleotides SSRs were 8 in the *S. Cusia*.

SSRs were also found in SSC region most prominently as mononucleotides and trinucleotides. For example, in *A. marina* 24 mononucleotides SSRs were found, *A. paniculata* 26 mononucleotides SSRs, *E. longzhouensis* 25 mononucleotides SSRs, *E. attenuates* 24 mononucleotides SSRs, *J. flava* 22 mononucleotides SSRs, *E. lofouensis*,* E. longipes J. leptostachya and S. Cusia* were fund to have 21 mononucleotides SSRs each. In *A. marina* no trinucleotide SSRs were found, however only few species were found to have 1 or 2 trinucleotides SSRs (Fig. 3C).

### Repeat sequence analysis

**Figure 4:**
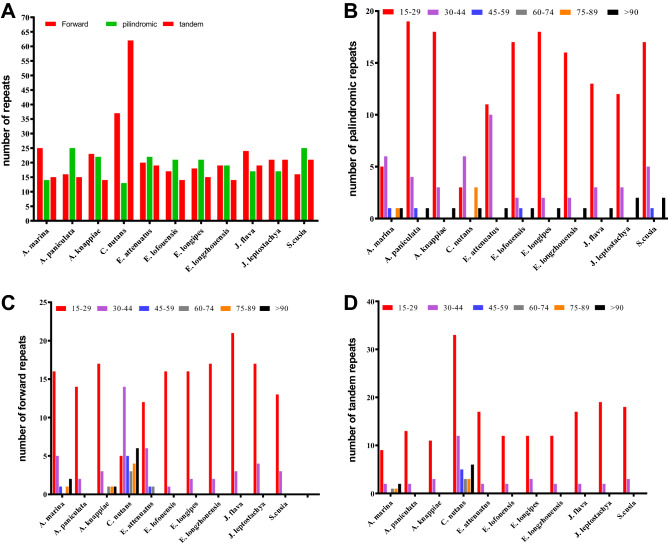
Distribution and frequency analysis of repeated sequences in chloroplast genome of *A. marina.* (**A**) total number of forward, palindromic and tandem repeats. (**B**) distribution of palindromic repeats in different ranges of length. (**C**) distribution of forward repeats in different ranges of length. (**D**) distribution of tandem repeats in different ranges of length.

In the chloroplast genome of *A. marina* and related species forward, reverse, palindromic and tandem repeats were analyzed. Total of 25 forward repeats, 14 palindromic repeats and 15 tandem repeats were found in *A. marina* cp genome. Total of 16, 23, 37, 20, 17, 18, 19, 24, 21 and 16 forward repeats were found in *A. paniculata*,* A. knappiae*,* C. nutans*,* E. attenuates*,* E. lofouensis*,* E. longipes*,* E. longzhouensis*,* J. flava*,* J. leptostachya* and *S. cusia* respectively (Fig. 4). Furthermore, tandem repeats in these species were 15, 14, 62, 19, 14, 15, 14, 19, 21 and 21 respectively. Among these repeats in *A. marina*, 16 of the forward repeats were found in the 15–29 bp, 5 repeats in 30–44 bp, 1 repeat was found in 45–59 bp, 75–79 bp and 2 repeats were found in > 90. Similarly, in the tandem repeats, 9 repeats were found in 15–29 bp, 2 repeats were found in 30–44 bp, 1 repeat was found in 60–74 bp, and 75–79 bp and 2 repeats in > 90 bp. Among the palindromic repeats, 5 repeats were found in the 15–29 bp, 6 repeats were found in 30–44 bp, and 1 repeat was found in 45–59 bp, 75–79 bp and > 90 bp.

The highest number of forward repeats (37) were found in the *C. nutans* and the lowest number of forward repeats (16) were found in the *A. paniculate* and *S. cusia.* Similarly, the highest tandem repeats (62) and the lowest number of tandem repeats (14) were found in the *A. knappiae*,* E. lofouensis* and *E. longzhouensis*. Additionally, among all the species, the highest number of palindromic repeats (25) were found in *A. paniculate* and *S. cusia* and the lowest palindromic repeats (14) were found in the *A. marina* (Fig. 4A–D).

### Contraction and expansion of IR region

**Figure 5:**
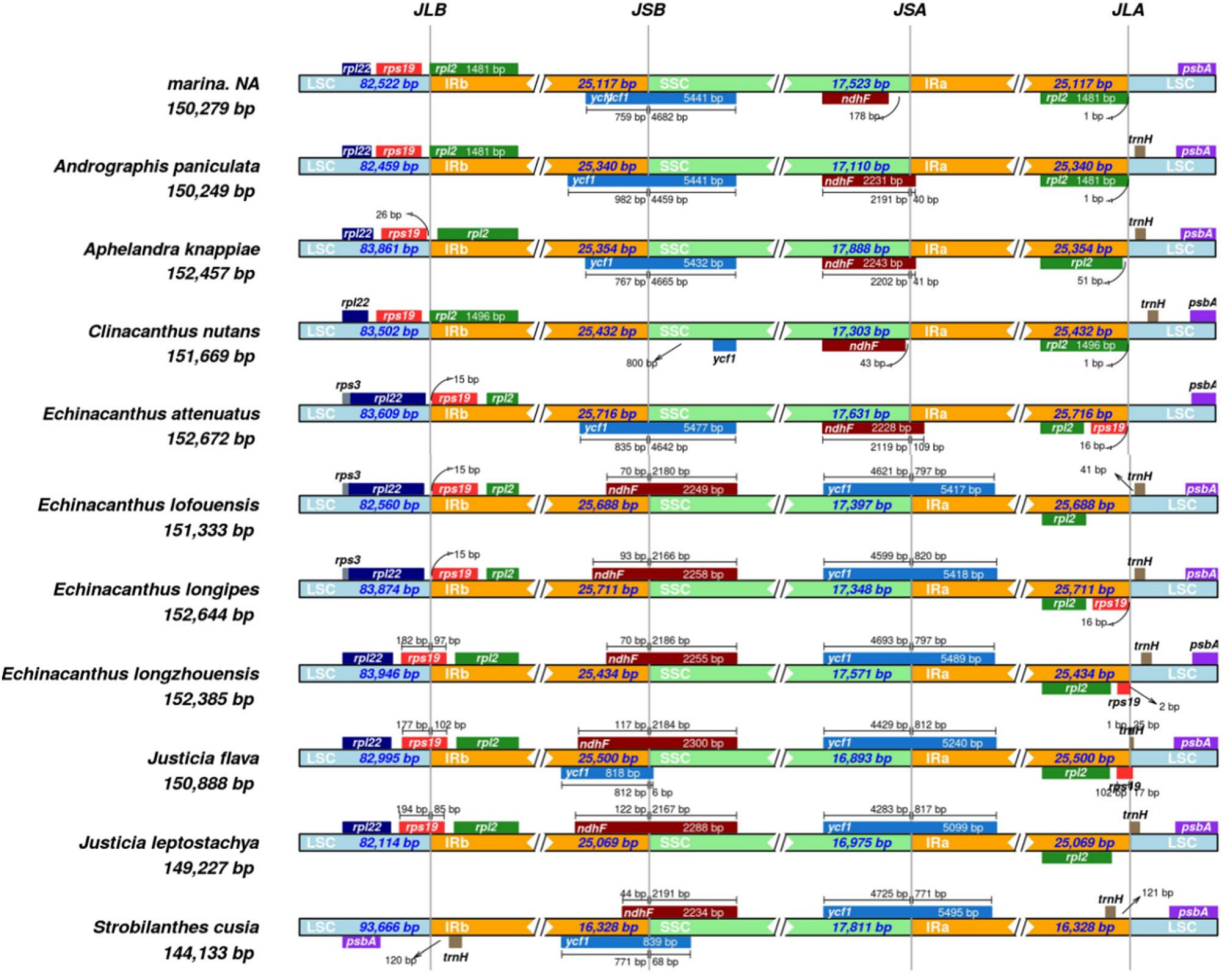
Distance between the adjacent genes and junctions of Large single Copy (LSC), two inverted repeats (IR) and Small Single Copy (SSC) in A. marina and 10 other related species. boxes below and above the lines represent the genes located on the border of LSC, IR and SSC. The figure represents the changes in the length and location of certain genes across these borders.

In the current study, contraction, and expansion in the 4-junctions (JSA, and JSB, JLA, JLB) between IRa and IRb, LSC and SSC regions of *A. marina* and 10 related species from the family Acanthaceae were analyzed. Size of IR regions in all the species were found almost same (25,000 bp to 25,700 bp) except in the specie *S. cusia* in which the IR region was found significantly lower (16,328 bp) as compared to the other species (Table 1). Some of the genes present on the borders of IR, SSC and LSC region were duplicated. For example, at JLB border, the *rps19* gene was found in the LSC region in the species *A. marina*,* A. paniculata*,* A. knappiae* and *C. nutans* (Fig. 5). However, this *rps19* gene was found in the IRb region about 15 bp away form JLB border in *E. attenuatus*,* E. lofouensis* and *E. longipes*, 19 bp in *E. longipes*, 97 bp in *E. longzhouensis*, 102 bp in *J. flava* and 85 bp in the *J. leptostachya*. Similarly, the gene ndhF was found partly extended in the IRb regions in some species such as *E. lofouensis* 70 bp, *E. longipes* 93 bp, *E. longzhouensis* 70 bp, J. flava 117 bp, *J. leptostachya* 122 bp and *S. cusia* 44 bp extended in the IRb region at JLB junction. However, the same gene (*ndhF*) was found in the SSC region in *A. marina* about 78 bp away for JSA border. On the other hand, this *ndhF* gene is partly extended to IRa region in some species where it is located about 40 bp, 41 bp 43 bp and 109 bp in the IRa regions in (*A. paniculate*, *A. knappiae*,* C. nutans* and *E. attenuatus*). Additionally, the *ycf1* gene was found at the IRs and SSC border in *E. lofouensis*, *E. longipes*, *E. longzhouensis*, *J. flava*, *J. leptostachya* and *S. cusia* were it extended to IRa region with (797, 820, 797, 812, 817 and 771) bp respectively (Fig. 5).

### Phylogenetic relationships among *A. marina* and related species

**Figure 6:**
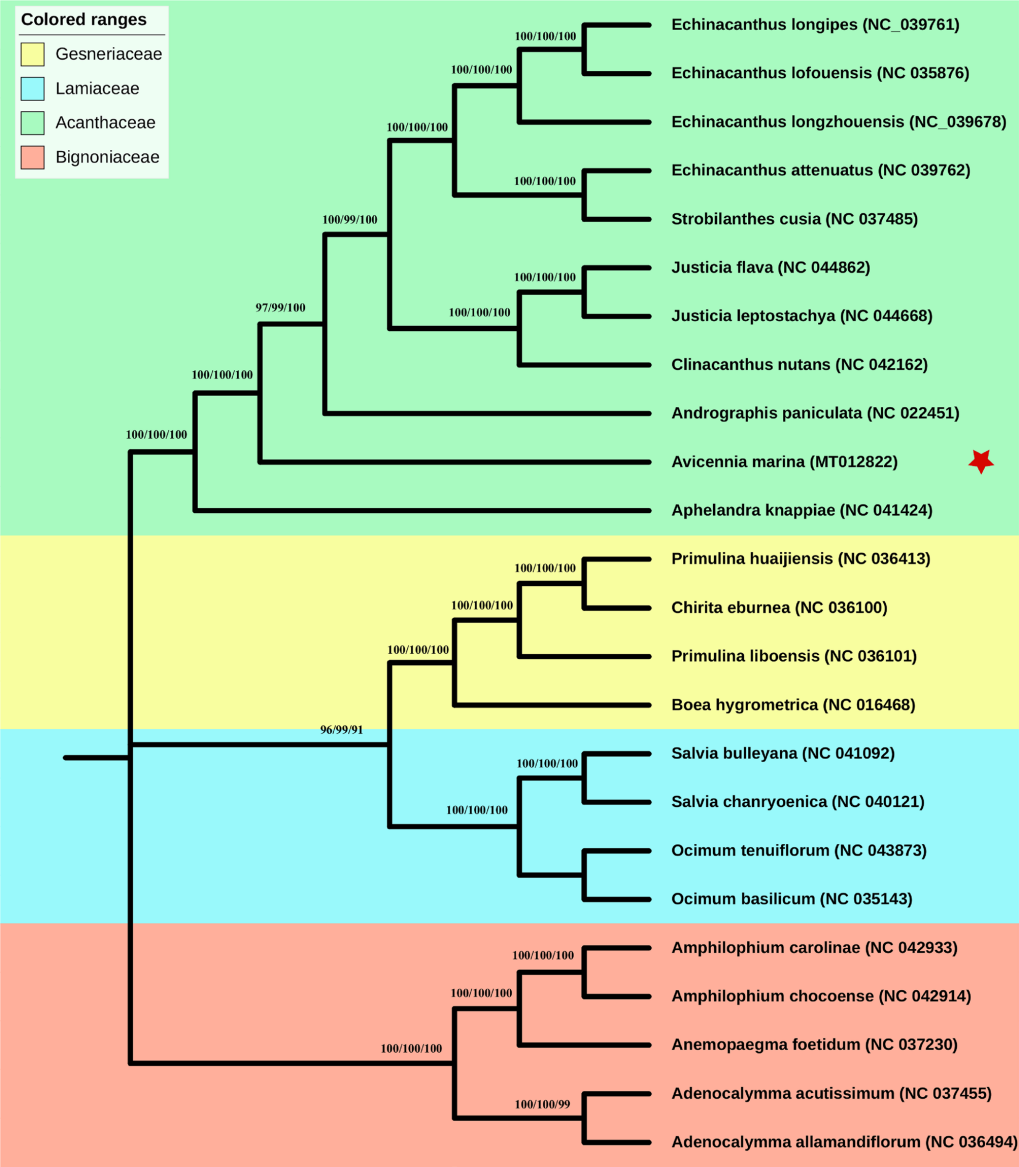
Phylogenetic trees were constructed for twenty-four species from four families using three different methods maximum likelihood (ML), maximum parsimony (MP) and neighbor -joining (NJ) by using 65 shared genes. Numbers above the branches are the bootstrap values of ML, MP and NJ respectively. Red star represents the position for *Avicennia marina*.

To determine the phylogenetic position of the *A. marina*, complete chloroplast genome was performed and compared with 24 other related species to build the phylogenetic tree. The phylogenetic position of *A. marina* is established using maximum likelihood (ML), maximum persimony (MP) and neighbour-jouining (NJ) methods in this study by utilizing 65 shared genes and complete cp genomes of related plant species. The phylogenetic trees constructed on three different methods shows same result and *A. marina* formed a clade near to *A. paniculata* and *A. knappiae* genomes from same family Acanthaceae. Similarly, Gesneriaceae was found the closest family to Acanthaceae (Fig. 6; Figure S2).

## Discussion

*A. marina* belongs to the family Acanthaceae which consist of more than 400 species distributed around the world^35^. The phylogenetic analysis is very important to correctly identify the taxonomic position of plants. Recently chloroplast genome sequencing has played a major role in the phylogenetic analysis of plants^36,37^. Though the chloroplast genomes are highly conserved when the size and genomic architecture are considered, however, the genes located on the borders of IR/SC varies tremendously in terms of size and type which make chloroplast distinctive for phylogenetic analysis^38,39^. The chloroplast genome sequence of all the 10 related species were also found conserved, however when compared with each other and with the chloroplast genome of *A. marina*, differences were observed for the genes located on the borders of IR/SC regions.

The size of complete chloroplast genome of *A. marina* (150,279 bp) is greater than the previously reported *J. laptostachya* (149,227 bp)^40^ and *S. Cusia* (144,133 bp)^41^, and almost same size of *A. paniculata* (150,249 bp)^42^ and *J. flava* (150,888)^43^. However, the size of *A. marina* chloroplast genome that we sequenced was found lower than the previously reported chloroplast genomes of the related species such as *A. knappiae*^44^, *C. nutans*^45^, *E. attenuates* , *E. lofouensis* , *E. longipes*^46^ and *E. longzhouensis*^43^. Our study also confirmed the LSC, SSC and IR region that were almost similar in size to the previously reported chloroplast genomes of *Eucalyptus globulus*^47^, *Coffea arabica* L.^48^ and *Camellia japonica* L.^49^. In our study, we have found that the GC contents (38.6%) of *A. marina* and on the 3rd position of codons, the GC contents (32.25%) were found lower as compared to AT/U contents (67.8%) which support previous reports of chloroplast genome sequences of *C. gileadensis* (37.9%) and *C. foliacea* (37.8%)^50^. The intron containing genes (14 genes) were found in which only 2 genes were found to contain two introns. The introns are very important in the gene expression regulations studies and it has been observed that when present on specific sites/positions, it can positively regulate exogeneous gene expression^51^. Thus, introns can be valuable tools in order to improve the transformation efficiencies. The intron sequences of chloroplast DNA also has key role in the phylogenetic analysis^52^.

Similarly, the size of protein coding genes (79,321 bp), rRNA (9,054 bp), tRNA (2,839 bp) and the number of genes (132), protein coding genes (87), rRNA (8) and tRNA (37) were found in our study that coincides with the previously reported studies on chloroplast genomes^50^. For example, in the same family (Acanthaceae), Ding, et al.^42^ and Huang, et al.^44^ previously reported the size of protein coding genes, rRNA, tRNA, number of genes, number of protein coding genes, number of rRNA and number of tRNA in *A. paniculate* and *A. knappiae* which is similar to what we found in the *A. marina*. Similarly, almost same results were observed in the recently reported chloroplast genomes of *C. gileadensis*^50^, *Teucrium species*^53^, *Vachellia nilotica* and *Senegalia senegal*^54^.

In the chloroplast genome, microsatellites or SSRs distributed in the genome with the length of almost 1 bp to 6 bp. Previously many studies reported SSRs at different positions in chloroplast genomes^55,56^. In the present study, SSRs distributed at different locations such as IR regions, LSC and SSC region were identified in the *A. marina*. Total of 126 SSRs were identified in *A. marina* and almost similar number of SSRs were found in the chloroplast genomes of related species such as *E. Longipes* (122) and *E. longzhouensis* (120). Other studies on chloroplast genomes have also confirmed the presence of uneven numbers of SSRs in the chloroplast genomes at different locations^50,57–59^.

Additionally, the forward, tandem and palindromic repeats in the *A. marina* were analyzed. The number of forward repeats were found 25, reverse repeats 11, palindromic repeats 14 and 15 tandem repeats were found in *A. marina*. The number of forward, reverse, tandem and palindromic repeats were found almost similar in the chloroplast genomes of the related species such *A. paniculate*^42^ and^44^. The pairwise alignment of gene in *A. marina* showed divergence of various genes in the coding regions as well as in the intergenic regions. The most divergent genes include rps16, *rpoC1*,* rpoC2*,* psbH*,* petB and accD*. Similarly the most prominent divergence in the intergenic region was found in *psbA-matK*,* rps16-psbL*,* atpA-atpF*,* atpH-atpI*,* rpoC1-rpoB*,* petN-psbM*,* psbD-rps14*,* ycf3—rps4*,* atpE-rbcL*,* accD-ycf4*,* psbL-petL*,* rps12-psbN*,* petD-rpl36*,* rpoA-rps11*,* rpl22-rps8*,* rpl16-rps3*,* ycf15-ndhB*,* rps19-rpl23*,* ndhF-ccsA*,* ndhD-psaC*,* ndhI-ndhG*,* ndhA-ycf1* and *ndhB-ycf2.* The gene divergence previously studied^52,60–62^ in the chloroplast genomes strongly confirms our findings of gene divergence.

The difference in the size of chloroplast genome is considered a common evolutionary practice which can be attributed to the contraction and expansion of the inverted repeats (the most conserved regions) in the genome^63^. In majority of the plants, the junctions or borders of the genome particularly in the quadripartite structure are very conserved. However, inversion at the borders or junction can be found in some species as previously reported^64^ as well as the loss of genes and the expansion and contraction which is the common cp genome event in the angiosperms^65,66^. The results in our study shows that *rps19* gene at JLB junction in *A. marina* has extended to IRb (15 to 102 bp) while the gene *ndhF* partly on the IRa region (40 to 178 bp) shows similar results patterns as previously studied by Cheon, et al.^67^.

The phylogenetic analysis is very important for the evolutionary and taxonomic studies. Many phylogenetic analysis are now based on the chloroplast genomes^68,69^. Previously, the phylogenetic analysis of mangrove (*A. marina*) were mostly based on the RAPD and other molecular markers^70,71^. Sahu, et al.^72^ reported that the role of multiple gene in the mangrove phylogeny. However, in this study we used both whole cp genomes and concatenated 65 protein coding genes to infer the phylogenetic position of *A. marina*. Both data sets showed same results and *A. marina* is closely related to *A. paniculate* and *A. knappiae* in family Acanthaceae. The present study provides a valuable analysis of the complete plastome of *A. marina* and related species, which may facilitate species identification and both biological and phylogenetic studies.

## Conclusion

Complete chloroplast genome sequence of *A. marina* was found highly conserved in its structure and order of genes distribution as compared to the other mangrove species in Acanthaceae. The results showed location and distribution of SSRs as well as the sequence divergence among the chloroplast genome of *A. marina* and related mangrove species. Among genes, *rpl22*,* ndhF*,* rps15* and *ndhA* were found the most divergent genes in the mangrove species. Additionally, the phylogenetic analysis shows that *A. marina* was closer to *A*. *knappiae* and *A. paniculate* species. It can be concluded from this study that complete chloroplast genome sequence may provide a better understanding of identification and phylogenetic studies of plant as compared to other strategies.

## Materials and methods

### Plant sample, Chloroplast DNA extraction and sequencing

*A. marina* seedlings were donated by the Center for Marine Conservation, Ministry of Environment, Sultanate of Oman. After shipping it to the greenhouse the leaves were collected in liquid nitrogen and ground to a fine powder. The powder samples of leaves were processed for chloroplast DNA extraction. Leaves were collected from the mangrove plants and were ground into fine powder using liquid nitrogen. Chloroplast DNA was extracted using the protocol of Khan et al.^73^. Manufacturer's instructions (Life Technologies, Carlsbad, CA, USA) were followed for the preparations of genomic libraries. Ion Shear Plus Reagents kit was used to shear chloroplast DNA into 400 bp fragments while Ion Xpress Plus gDNA Fragment Library kit was used for library preparation. Quantity and quality of libraries were checked using the Qubit 3.0 fluorometer and Bioanalyzer (Agilent 2100 Bioanalyzer system; Life Technologies, Carlsbad, CA, USA). The template was amplified using Ion 520 & 530 OT2 Reagents and enriched by using the OneTouch instrument and Ion OneTouch ES enrichment system. The final chloroplast DNA sample was loaded into Ion S5 Sequencing (supported by Mawarid, Oman’s Animal & Plant Genetic Resource Center, Ministry of Higher Education, Researcher & Innovation, Oman) Chip followed by sequencing through the Ion Torrent S5 protocol^34,53^.

### Genome assembly and annotation

A total of 2,256,283, raw reads were generated for the chloroplast genome of *A. marina*. The cp genome of *A. marina* was mapped with other chloroplast genomes such as *Andrographis paniculate*^74^, using Bowtie2 (v.2.2.3)^75^ in Geneious Pro (v.10.2.3) software^76^. For the assembly, the mean coverage of *A. marina* was 99X. IR region was identified by using the MITObim (v.1.8) software^50,77^ was used for the sequence length adjustment. FASTQC^78,79^ was performed after sequencing to check the read quality. For biases reduction in the analysis, an in-house script was used to filter reads if less than 90% of the bases that made up the read were below Q20. Adopter sequences were removed using the Trimmomatic (v0.36)^80^. Bowtie2 in Geneious Pro (v.10.2.3)^76^ were used for mapping only high quality reads.

Dual Organellar Genome Annotator (DOGMA)^81^ was used for the genome annotation. For the position’s identification of transfer and ribosomal RNA and coding genes BLASTX and BLASTN were used. Furthermore, tRNAscan-SE version 1.21^82^ was used for the tRNA gene annotation. In order to compare the cp genome of *A. marina* with the related cp genomes, tRNAscan-SE and Geneious software were used. Intron boundaries, stop and start codons were adjusted by comparing it to the previously reported cp genome of the related specie from family Acanthaceae. OGDRAW^83^ was used for the illustrations of structural features of *A. marina* cp genome. MEGA-X software^84^ was used for the determination of deviations in synonymous codon usage and relative synonymous codon usage with amino acid composition influence. The pairwise gene divergence was determined by mVISTA^85^ in Shuffle-LAGAN mode.

### Identification of repetitive sequences and SSRs

The REPuter software^86^ was used for the identification of forward, reverse and palindromic repeats, the sequence identity with 90% and minimum 15 bp was the basic criteria in the identification. Microsatellite analysis of contig sequences was carried out with the MIcroSAtellite (MISA) identification tool^87^. The parameters (unit_size, min_repeats) were defined as follows: 1–10, 2–8, 3–4, 4–4, 5–3, and 6–3; the minimum distance between two SSRs was set to 100. For the identification of tandem repeats, Tandem Repeat Finder version 4.087^88^ b was used for tandem repeat identification.

### Sequence divergence and Phylogenetic analysis

The pairwise distance among the cp genes shared by *A. marina* and related species as well as the complete cp genome of *A. marina* was determined. The ambiguous and missing genes annotation were identified and removed by the comparative sequence analysis. Complete cp genomes were aligned by using the MAFFT version 7.222^89^ and Kimura's two-parameter (K2P) model^90^ was used for calculation of pairwise sequence distance. The divergence of the new *A. marina* cp genomes from related species of family Acanthaceae was assessed using mVISTA^85^ in Shuffle-LAGAN mode and by employing the new *A. marina* genome as reference.

For the determination of phylogenetic position of *A. marina*, 23 published cp genomes were downloaded from NCBI from four different families (Gesnerianceae, Lamiaceae, Bignoniaceae and Acanthaceae) respectively. Complete cp genomes and a separate partition containing only the 65 shared genes (concatenated) were used to infer the phylogenetic position of *A*. *marina*. MAFFT version 7.222^89^, with default parameters were used for the alignment of both complete genomes and shared genes data sets. Maximum likelihood (ML) and neighbour-joining (NJ) methods were used to infer the phylogenetic trees with MEGA-X^91^ and parameters were adjusted with a BIONJ tree with 1000 bootstrap replicates using the Kimura 2-parameter model with gamma-distributed rate heterogeneity and invariant sites. Maximum parsimony (MP) by using PAUP^92^ using previously described settings^33,93^.

## Supplementary Information

Supplementary Information 1.

Supplementary Information 2.

## Data Availability

All data generated or analyzed during this study are included in this published article.

## References

[1] Faridah-Hanum I (2019). Development of a comprehensive mangrove quality index (MQI) in Matang Mangrove: assessing mangrove ecosystem health. Ecol. Ind..

[2] Spalding M (2010). World Atlas of Mangroves.

[3] Himes-Cornell A, Grose SO, Pendleton L (2018). Mangrove ecosystem service values and methodological approaches to valuation: where do we stand?. Front. Mar. Sci..

[4] Wu Y, Ricklefs RE, Huang Z, Zan Q, Yu S (2018). Winter temperature structures mangrove species distributions and assemblage composition in China. Glob. Ecol. Biogeogr..

[5] Das SS, Das S, Ghosh P (2015). Phylogenetic relationships among the mangrove species of Acanthaceae found in Indian Sundarban, as revealed by RAPD analysis. Adv. Appl. Sci. Res.

[6] Kathiresan K, Bingham BL (2001). Biology of mangroves and mangrove ecosystems. Adv. Mar. Biol..

[7] Sannigrahi S (2020). Responses of ecosystem services to natural and anthropogenic forcings: a spatial regression based assessment in the world's largest mangrove ecosystem. Sci. Total Environ..

[8] dos Santos NM, Lana P (2017). Present and past uses of mangrove wood in the subtropical Bay of Paranaguá (Paraná, Brazil). Ocean Coast. Manag..

[9] Jusoff K (2013). Malaysian mangrove forests and their significance to the coastal marine environment. Pol. J. Environ. Stud..

[10] Duke NC, Lo E, Sun M (2002). Global distribution and genetic discontinuities of mangroves–emerging patterns in the evolution of Rhizophora. Trees.

[11] Nagelkerken I (2008). The habitat function of mangroves for terrestrial and marine fauna: a review. Aquat. Bot..

[12] Tomlinson PB (2016). The Botany of Mangroves.

[13] Dahdouh-Guebas F (2005). How effective were mangroves as a defence against the recent tsunami?. Curr. Biol..

[14] Saenger P (2002). Mangrove Ecology, Silviculture and Conservation.

[15] Lakshmi M, Parani M, Parida A (2002). Molecular phylogeny of mangroves IX: molecular marker assisted intra-specific variation and species relationships in the Indian mangrove tribe Rhizophoreae. Aquat. Bot..

[16] Grover A, Sharma P (2016). Development and use of molecular markers: past and present. Crit. Rev. Biotechnol..

[17] Adsul GG, Chaurasia AK, Dhake AV, Kothari RM (2015). RAPD analysis of phylogenetic relationships and genetic variations in genus Allium. Biochem. Indian J..

[18] Xu K (2016). Identification of tuna species (*Thunnini tribe*) by PCR-RFLP analysis of mitochondrial DNA fragments. Food Agric. Immunol..

[19] Wambugu PW, Brozynska M, Furtado A, Waters DL, Henry RJ (2015). Relationships of wild and domesticated rices (Oryza AA genome species) based upon whole chloroplast genome sequences. Sci. Rep..

[20] Middleton CP (2014). Sequencing of chloroplast genomes from wheat, barley, rye and their relatives provides a detailed insight into the evolution of the Triticeae tribe. PLoS ONE.

[21] Raman G, Park S (2016). The complete chloroplast genome sequence of Ampelopsis: gene organization, comparative analysis, and phylogenetic relationships to other angiosperms. Front. Plant Sci..

[22] Yang JB, Li DZ, Li HT (2014). Highly effective sequencing whole chloroplast genomes of angiosperms by nine novel universal primer pairs. Mol. Ecol. Resour..

[23] Su H-J, Hogenhout SA, Al-Sadi AM, Kuo C-H (2014). Complete chloroplast genome sequence of Omani lime (*Citrus aurantiifolia*) and comparative analysis within the rosids. PLoS ONE.

[24] Hu, S. *Phylogeny and Chloroplast Evolution in BRASSICACEAE*, University of Trento, (2016).

[25] Santos, C. G. Development of new tools for the identification of plants using chloroplast DNA sequences. (2018).

[26] Singh BP, Kumar A, Kaur H, Singh H, Nagpal AK (2020). CpGDB: A comprehensive database of chloroplast genomes. Bioinformation.

[27] Wu Z (2015). The new completed genome of purple willow (Salix purpurea) and conserved chloroplast genome structure of Salicaceae. J. Nat. Sci.

[28] Wu Z (2016). The whole chloroplast genome of shrub willows (Salix suchowensis). Mitochondrial DNA Part A.

[29] Egamberdiev SS (2016). Comparative assessment of genetic diversity in cytoplasmic and nuclear genome of upland cotton. Genetica.

[30] Williams AV, Miller JT, Small I, Nevill PG, Boykin LM (2016). Integration of complete chloroplast genome sequences with small amplicon datasets improves phylogenetic resolution in Acacia. Mol. Phylogenet. Evol..

[31] Fučíková K (2014). New phylogenetic hypotheses for the core Chlorophyta based on chloroplast sequence data. Front. Ecol. Evol..

[32] Friis, G. *et al.* A high-quality genome assembly and annotation of the gray mangrove, Avicennia marina. *bioRxiv* (2020). 10.1093/g3journal/jkaa025 PMC8022769 33561229

[33] Asaf S (2016). Complete chloroplast genome of Nicotiana otophora and its comparison with related species. Front. Plant Sci..

[34] Asaf S, Khan AL, Khan A, Al-Harrasi A (2020). Unraveling the chloroplast genomes of two prosopis species to identify its genomic information, comparative analyses and phylogenetic relationship. Int. J. Mol. Sci..

[35] Souladeth P, Tagane S, Zhang M, Okabe N, Yahara T (2017). Flora of Nam Kading National Protected Area I: a new species of yellow-flowered Strobilanthes (Acanthaceae), *S*.* namkadingensis*. PhytoKeys.

[36] Yang L (2020). The complete chloroplast genome of Swertia tetraptera and phylogenetic analysis. Mitochondrial DNA Part B.

[37] Biju VC (2019). The complete chloroplast genome of *Trichopus zeylanicus*, and phylogenetic analysis with dioscoreales. Plant Genome.

[38] Silva SR (2016). The chloroplast genome of *Utricularia reniformis* sheds light on the evolution of the ndh gene complex of terrestrial carnivorous plants from the Lentibulariaceae family. PLoS ONE.

[39] Zuo L-H (2017). The first complete chloroplast genome sequences of Ulmus species by de novo sequencing: Genome comparative and taxonomic position analysis. PLoS ONE.

[40] Niu Z, Huang S, Deng Y, Chen X (2019). The complete chloroplast genome of Justicia leptostachya (Acanthaceae). Mitochondrial DNA Part B.

[41] Chen H (2018). Sequencing and analysis of Strobilanthes cusia (Nees) Kuntze chloroplast Genome revealed the rare simultaneous contraction and expansion of the inverted repeat region in Angiosperm. Front. Plant Sci..

[42] Ding P (2016). The complete chloroplast genome sequence of the medicinal plant Andrographis paniculata. Mitochondrial DNA Part A.

[43] Yaradua SS, Alzahrani DA, Albokhary EJ, Abba A, Bello A (2019). Complete chloroplast genome sequence of Justicia flava: genome comparative analysis and phylogenetic relationships among Acanthaceae. BioMed Res. Int..

[44] Huang S, Deng Y, Ge X (2019). The complete chloroplast genome of Aphelandra knappiae (Acanthaceae). Mitochondrial DNA Part B.

[45] Li M-N (2019). Complete plastome sequence of Clinacanthus nutans (Acanthaceae): a medicinal species in Southern China. Mitochondrial DNA Part B.

[46] Jiang M, Wang J, Zhang H (2020). Characterization and phylogenetic analysis of the complete chloroplast genome sequence of *Disanthus cercidifolius* subsp. longipes (Hamamelidaceae), a rare and endangered wild plant species in China. Mitochondrial DNA Part B.

[47] Steane DA (2005). Complete nucleotide sequence of the chloroplast genome from the Tasmanian blue gum, *Eucalyptus globulus* (Myrtaceae). DNA Res..

[48] Park J, Kim Y, Xi H, Heo K-I (2019). The complete chloroplast genome of ornamental coffee tree, *Coffea arabica* L. (Rubiaceae). Mitochondrial DNA Part B.

[49] Park J (2019). The complete chloroplast genome of common camellia tree, *Camellia japonica* L. (Theaceae), adapted to cold environment in Korea. Mitochondrial DNA Part B.

[50] Arif Khan SA (2019). First complete chloroplast genomics and comparative phylogenetic analysis of *Commiphora gileadensis* and *C. foliacea*: Myrrh producing trees. PLoS ONE.

[51] Xu J (2003). The first intron of rice EPSP synthase enhances expression of foreign gene. Sci. China Ser. C Life Sci..

[52] Kelchner SA (2000). The evolution of non-coding chloroplast DNA and its application in plant systematics. Ann. Missouri Bot. Gard..

[53] Khan A (2019). Complete chloroplast genomes of medicinally important Teucrium species and comparative analyses with related species from Lamiaceae. PeerJ.

[54] Asaf S, Khan A, Khan AL, Al-Harrasi A, Al-Rawahi A (2019). Complete chloroplast genomes of *Vachellia nilotica* and *Senegalia senegal*: comparative genomics and phylogenomic placement in a new generic system. PLoS ONE.

[55] Su Y, He Z, Wang Z, Hong Y, Wang T (2019). Characterization of the complete chloroplast genome of Leptochilus decurrens (Polypodiaceae), a least concern folk medicinal fern. Mitochondrial DNA Part B.

[56] Kumar S, Shanker A (2020). In silico comparative analysis of simple sequence repeats in chloroplast genomes of genus nymphaea. J. Sci. Res..

[57] Asaf S (2017). The complete chloroplast genome of wild rice (Oryza minuta) and its comparison to related species. Front. Plant Sci..

[58] Asaf S (2018). Complete chloroplast genome sequence and comparative analysis of loblolly pine (*Pinus taeda* L.) with related species. PLoS ONE.

[59] Khan AL (2017). The first chloroplast genome sequence of Boswellia sacra, a resin-producing plant in Oman. PLoS ONE.

[60] Asaf S (2017). Comparative analysis of complete plastid genomes from wild soybean (*Glycine soja*) and nine other Glycine species. PLoS ONE.

[61] Qian J (2013). The complete chloroplast genome sequence of the medicinal plant *Salvia miltiorrhiza*. PLoS ONE.

[62] Wang W, Messing J (2011). High-throughput sequencing of three Lemnoideae (duckweeds) chloroplast genomes from total DNA. PLoS ONE.

[63] Shen X (2017). Complete chloroplast genome sequence and phylogenetic analysis of the medicinal plant *Artemisia annua*. Molecules.

[64] Liu M (2016). The complete chloroplast genome sequence of Tartary Buckwheat Cultivar Miqiao 1 (Fagopyrum tataricum Gaertn.). Mitochondrial DNA Part B.

[65] Fu P-C, Zhang Y-Z, Geng H-M, Chen S-L (2016). The complete chloroplast genome sequence of *Gentiana lawrencei* var. farreri (Gentianaceae) and comparative analysis with its congeneric species. PeerJ.

[66] Choi KS, Chung MG, Park S (2016). The complete chloroplast genome sequences of three Veroniceae species (Plantaginaceae): comparative analysis and highly divergent regions. Front. Plant Sci..

[67] Cheon K-S, Kim K-A, Kwak M, Lee B, Yoo K-O (2019). The complete chloroplast genome sequences of four Viola species (Violaceae) and comparative analyses with its congeneric species. PLoS ONE.

[68] Munyao JN (2020). Complete chloroplast genomes of chlorophytum comosum and chlorophytum gallabatense: genome structures, comparative and phylogenetic analysis. Plants.

[69] Yu, T., Huang, B.-H., Zhang, Y., Liao, P.-C. & Li, J.-Q. Chloroplast genome of an extremely endangered conifer Thuja sutchuenensis Franch.: gene organization, comparative and phylogenetic analysis. *Physiol. Mol. Biol. Plants* 1–10 (2020). 10.1007/s12298-019-00736-7 PMC7078402 32205919

[70] Sabri DM, El-Hussieny SA, Elnwishy N (2018). Genotypic Variations of Mangrove (Avicennia marina) in Nabq Protectorate, South Sinai Egypt. Int. J. Agric. Biol..

[71] 71Basyuni, M., Baba, S. & Oku, H. in *IOP Conference Series: Materials Science and Engineering.* 012247 (IOP Publishing).

[72] Sahu SK, Singh R, Kathiresan K (2016). Multi-gene phylogenetic analysis reveals the multiple origin and evolution of mangrove physiological traits through exaptation. Estuar. Coast. Shelf Sci..

[73] Khan A (2019). First complete chloroplast genomics and comparative phylogenetic analysis of *Commiphora gileadensis* and *C. foliacea*: Myrrh producing trees. PLoS ONE.

[74] Arif MF, Aristya GR, Subositi D, Sari AN, Kasiamdari RS (2019). rbcL and matK chloroplast DNA composition of green chireta (*Andrographis paniculata*) from Indonesia. Biodivers. J. Biol. Divers..

[75] Langdon WB (2015). Performance of genetic programming optimised Bowtie2 on genome comparison and analytic testing (GCAT) benchmarks. BioData Min..

[76] Kearse M (2012). Geneious Basic: an integrated and extendable desktop software platform for the organization and analysis of sequence data. Bioinformatics.

[77] Gan HM, Schultz MB, Austin CM (2014). Integrated shotgun sequencing and bioinformatics pipeline allows ultra-fast mitogenome recovery and confirms substantial gene rearrangements in Australian freshwater crayfishes. BMC Evol. Biol..

[78] Feng G, Yang J, Peng F-R (2020). Characterization of complete chloroplast genome of artificial hybrid passion fruit ‘Ziyan’, Passiflora edulis Sims× P. edulis f. edulis Sims (Passifloraceae). Mitochondrial DNA Part B.

[79] Brown J, Pirrung M, McCue LA (2017). FQC Dashboard: integrates FastQC results into a web-based, interactive, and extensible FASTQ quality control tool. Bioinformatics.

[80] Bolger AM, Lohse M, Usadel B (2014). Trimmomatic: a flexible trimmer for Illumina sequence data. Bioinformatics.

[81] Wyman SK, Jansen RK, Boore JL (2004). Automatic annotation of organellar genomes with DOGMA. Bioinformatics.

[82] Schattner P, Brooks AN, Lowe TM (2005). The tRNAscan-SE, snoscan and snoGPS web servers for the detection of tRNAs and snoRNAs. Nucleic Acids Res..

[83] Lohse M, Drechsel O, Bock R (2007). OrganellarGenomeDRAW (OGDRAW): a tool for the easy generation of high-quality custom graphical maps of plastid and mitochondrial genomes. Curr. Genet..

[84] Kumar S, Stecher G, Li M, Knyaz C, Tamura K (2018). MEGA X: molecular evolutionary genetics analysis across computing platforms. Mol. Biol. Evol..

[85] Frazer KA, Pachter L, Poliakov A, Rubin EM, Dubchak I (2004). VISTA: computational tools for comparative genomics. Nucleic Acids Res..

[86] Kurtz S (2001). REPuter: the manifold applications of repeat analysis on a genomic scale. Nucleic Acids Res..

[87] Beier S, Thiel T, Münch T, Scholz U, Mascher M (2017). MISA-web: a web server for microsatellite prediction. Bioinformatics.

[88] Wirawan, A., Kwoh, C. K., Hsu, L. Y. & Koh, T. H. in *International Conference on Computational Systems-Biology and Bioinformatics.* 151–164 (Springer).

[89] Katoh K, Standley DM (2013). MAFFT multiple sequence alignment software version 7: improvements in performance and usability. Mol. Biol. Evol..

[90] Srivathsan A, Meier R (2012). On the inappropriate use of Kimura-2-parameter (K2P) divergences in the DNA-barcoding literature. Cladistics.

[91] Kumar S, Nei M, Dudley J, Tamura K (2008). MEGA: a biologist-centric software for evolutionary analysis of DNA and protein sequences. Brief. Bioinf..

[92] Swofford, D. L. Paup*: Phylogenetic analysis using parsimony (and other methods) 4.0. B5. (2001).

[93] Wu Z, Tembrock LR, Ge S (2015). Are differences in genomic data sets due to true biological variants or errors in genome assembly: an example from two chloroplast genomes. PLoS ONE.

