# Supplementary material for: Mangrove tree (Avicennia marina): insight into chloroplast genome evolutionary divergence and its comparison with related species from family Acanthaceae
Source: Sci Rep. 2021 Feb 11;11:3586. doi: 10.1038/s41598-021-83060-z (PMC7878759; doi:10.1038/s41598-021-83060-z)
Supplement: Supplementary file 1 — Supplementary Information 1. [file 41598_2021_83060_MOESM1_ESM.docx]

**Mangrove tree (*Avicennia marina*): insight into chloroplast genome evolutionary divergence and its comparison with related species from family Acanthaceae**

Sajjad Asaf^1#^, Abdul Latif Khan^1#*^, Muhammad Numan^1^, Ahmed Al-Harrasi^1^*

^1^ Natural and Medical Sciences Research Center, University of Nizwa, Nizwa, Oman

**Short title: Comparative genomics of *Avicennia marina in* Acanthaceae**

**Corresponding Authors:**

Abdul Latif Khan ([latifepm78@yahoo.co.uk](mailto:latifepm78@yahoo.co.uk))

Ahmed Al-Harrasi ([aharrasi@unizwa.edu.om)](mailto:aharrasi@unizwa.edu.om))

#Authors equally contributed

**Supplementary Materials**

**S1 Fig.** **Visual alignment of plastid genomes from *A. marina* and related species from family Acanthaceae.** VISTA-based identity plot showing sequence identity among eleven species, using *A. marina* as a reference genome.

**S2 Fig.** Phylogenetic trees were constructed for twenty-four species from four families using three different methods maximum likelihood (ML), maximum parsimony (MP) and neighbor -joining (NJ) by using complete genome data set. Numbers above the branches are the bootstrap values of ML, MP and NJ respectively.

**
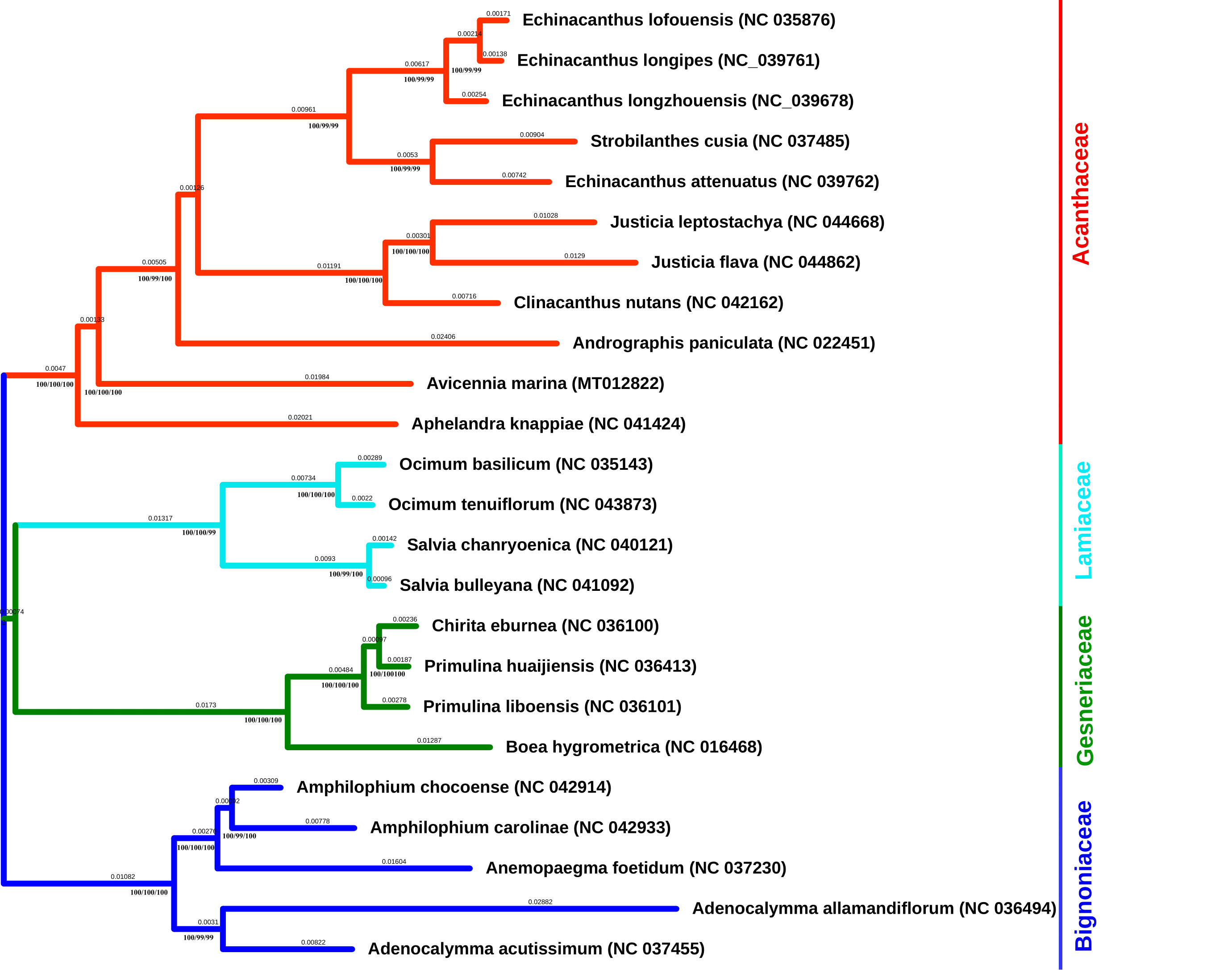
**

**Supplementary Data 1. Average pairwise sequence distance of *A. marina* with related species chloroplast genomes from family Acanthaceae**
